# Sustainable valorization of biogenic coral limestone waste into calcium aluminate biosorbents for efficient Cr(vi) remediation: characterization, experimental performance, and statistical physics analysis

**DOI:** 10.1039/d5ra09083k

**Published:** 2026-02-13

**Authors:** S. H. Kenawy, Mortaga M. Abou-Krisha, Tarek A. Yousef, Yasser F. Salama, Eder C. Lima, Glaydson S. dos Reis, Ahmed M. Salah, Moaaz K. Seliem

**Affiliations:** a Refractories, Ceramics and Building Materials Department, National Research Centre 33 El Bohouth Street, Dokki Giza Egypt; b Chemistry Department, College of Science, Imam Mohammad Ibn Saud Islamic University (IMSIU) Riyadh 11623 Saudi Arabia; c Geology Department, Faculty of Science, Beni-Suef University Beni Suef Egypt yasser.salama@science.bsu.edu.eg; d Institute of Chemistry, Federal University of Rio Grande do Sul (UFRGS) Av. Bento Goncalves 9500, Postal Box, 15003 Porto Alegre RS 91501-970 Brazil; e Laboratory of Industrial Chemistry and Reaction Engineering, Faculty of Science and Engineering, Åbo Akademi University 20500 Åbo/Turku Finland; f Faculty of Earth Science, Beni–Suef University Beni Suef 62511 Egypt

## Abstract

Herein, *Hydnophora* coral waste was chemically treated and thermally processed to form calcium aluminate ceramic (CAC), which served as an efficient adsorbent for Cr(vi) remediation. The produced CAC biosorbent was characterized using SEM-EDX, XRD, FTIR, and TEM techniques. Adsorption behavior was systematically evaluated through kinetic, equilibrium, thermodynamic, and statistical-physics modeling. Regeneration tests were performed to evaluate stability and reusability over multiple adsorption–desorption cycles. The developed CAC principally included crystalline mayenite (Ca_12_Al_14_O_33_) and Ca_4_Al_6_O_13_ phases, confirming successful phase transformation during the fabrication process. Kinetic analysis indicated that the pseudo-first-order model provided the best fit for the adsorption process, while the Liu isotherm most effectively described the equilibrium data. The as-synthesized CAC biosorbent exhibited notable Cr(vi) adsorption capacity (148.65–304.62 mg g^−1^ across 25–55 °C), reflecting a strong dependence on solution temperature. Thermodynamic analysis confirmed a spontaneous uptake, as Δ*G*° ranged from −5.064 to −7.528 kJ mol^−1^ at 298–328 K, and an endothermic process, as Δ*H*° was +19.601 kJ mol^−1^. The low Δ*E* values ranging from 11.45 kJ mol^−1^ (25 °C) to 13.11 kJ mol^−1^ (55 °C) indicated that the adsorption system is mainly governed by a physisorption mechanism (Δ*E* < 15 kJ mol^−1^). Statistical physics analysis revealed a horizontal multi-docking adsorption configuration, and steric parameters emphasized the significance of the functional group density of CAC in enhancing uptake performance. Additionally, the prepared material exhibited excellent regeneration capability, maintaining high performance over multiple adsorption–desorption cycles. Overall, this work highlights a sustainable method for converting coral limestone waste into high-performance ceramic adsorbents. Additionally, the use of statistical physics theory offers valuable insight into the molecular-level mechanisms controlling Cr(vi) adsorption.

## Abstract 2

Herein, *Hydnophora* coral waste was chemically treated and thermally processed to form calcium aluminate ceramic (CAC), which served as an efficient adsorbent for Cr(vi) remediation.

## Graphical abstract

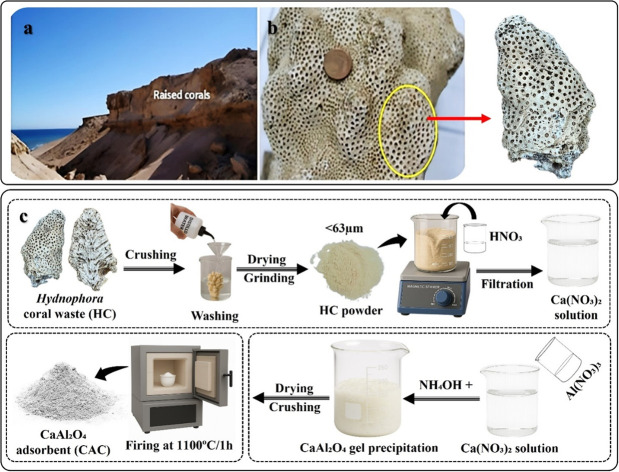

## 1. Introduction

Large volumes of metal ions are increasingly being released into the environment by a variety of industrial activities, including papermaking, leather tanning, electroplating, mining, and metal finishing processes.^1^ Continuous discharge of these metal ions into water bodies leads to severe water pollution, posing significant risks to human health and aquatic ecosystems.^2^ Many of these metals, including Cr(vi), Pb(ii), and Cd(ii), are highly toxic and non-biodegradable, and can accumulate in the human body or contaminate water sources, highlighting the urgent need for effective and sustainable methods to remove toxic metals from contaminated water bodies.^2^

The continuous release of chromium into water resources raises serious environmental concern, particularly because Cr(vi) persists as highly soluble oxyanions, mainly chromate and dichromate species such as CrO_4_^2−^, HCrO_4_^−^, and Cr_2_O_7_^2−^, whose distribution depends on pH and water chemistry.^1–4^ Significant environmental alarms result from the continuous release of chromium (Cr) ions into water resources.^3^ Hexavalent Cr(vi) and trivalent Cr(iii) are metal ions with dissimilar biological, chemical, and environmental properties.^4^ Compared to the trivalent form, hexavalent chromium is more easily soluble and moveable in solutions.^5^ Furthermore, the hexavalent form of chromium is 500 times more hazardous than the trivalent form and has been linked to a number of illnesses in humans, including lung cancer, liver damage, stomach ulcers, vomiting, and damage to nerve tissue.^6–8^ The maximum values of Cr(vi) in portable water and inland surface water were recognized to be 0.05 mg L^−1^ and 0.1 mg L^−1^, respectively.^9^

As compared to other approaches like ion-exchange, advanced oxidation, and biological treatments, numerous studies showed that the adsorption method was suggested as an efficient, simple, and inexpensive approach in water remediation.^4–8^ A number of substances have been listed as adsorbents to remove Cr(vi) from solutions, including farming wastes,^10^ a surfactant–silica gelatin composite,^11^ activated zeolite,^12^ modified biomass waste,^13^ activated rectorite,^14^ active carbon,^15^ spent clay,^16^ surfactant-modified clay,^17^ CTAB-modified coal,^18^ and a H_2_O_2_-activated anthracite/chitosan composite.^19^

Recent studies have highlighted that modifying material composition and surface chemistry can enhance metal-related applications in environmental systems, including both analytical monitoring and remediation.^20–22^ For instance, metal-oxide nanostructures were employed in the detection of hazardous contamination.^20,21^ Moreover, functional porous frameworks, including metal–organic frameworks (MOFs), demonstrate the roles of the engineered active sites and porosity in the reduction of CO_2_.^22^ In parallel, hybrid composites have been extensively used for the efficient removal of heavy-metal ions and organic dyes from aqueous solutions.^23–25^ These materials combine the advantages of their specific components, providing high surface area, porous structure, chemical stability, and reusability, making them highly effective for water treatment applications.^26,27^ Following the mentioned approaches, the present study valorizes *Hydnophora* coral biowaste by converting it into low-cost and eco-friendly calcium-aluminate ceramic phases for water remediation.

Earlier studies have shown that substances rich in carbonates retain strong potential for purifying water by removing diverse contaminants.^28–30^ The skeletons of many invertebrates, such as mollusks, corals, and echinoderms, are usually made of calcium carbonate (CaCO_3_).^31^ The calcareous rigid portion of invertebrates offers a sustainable alternative for a range of manufacturing and ecological applications due to its special properties, including its high strength, durability, biodegradability, and thermal resistance.^32^ Coral limestone, a naturally white and brittle material, often becomes environmental biowaste due to its tendency to damage easily during storms, generating significant tons of carbonate-rich biowaste.^33^ Consequently, utilizing coral as a sustainable material for water remediation has been proposed.^34^ For instance, glass debris and *Favia* coral limestone biowaste were recently employed to design an alkali-activated binder that was functionalized with CTAB surfactant to facilitate the elimination of Mn(vii) from aqueous environments.^35^

Calcium aluminate ceramic (CAC) is the term for fired ceramics based on calcium aluminates, which are compounds made from calcium oxide (CaO) and aluminum oxide (Al_2_O_3_). These ceramic products are characterized by their high-temperature resistance, chemical endurance, and unusual features like rapid strength growth at low temperatures, making them attractive in numerous medical and industrial applications.^36^ CAC was prepared by fusing or sintering alumina (Al_2_O_3_) with properly proportioned aluminous and calcareous minerals (*e.g.*, CaO or CaCO_3_) at temperatures higher than 1400 °C.^37,38^ As an adsorbent, calcium aluminate phase was developed *via* the combustion technique and used in removing fluoride ions from polluted solutions.^39^

The purpose of this study was to transform *Hydnophora* coral (HC) biowaste into calcium aluminate phases appropriate for Cr(vi) extraction from contaminated aqueous systems. To optimize the uptake process, the properties of significant experimental variables such as pH, adsorbent dose, and selected temperature were systematically investigated. Kinetics, simple isotherms, and thermodynamics parameters were also considered to evaluate the adsorption mechanism. Furthermore, the adsorption system was analyzed at the microscopic scale using a number of statistical physics equations, which enabled evaluation of steric and energetic interactions according to the most suitable theoretical fit. Overall, the present study offers a distinctive method that enhances the limestone corals biowaste towards the production of bio-based ceramic adsorbent for removing metallic ions from solutions.

## 2. Geological setting

The Red Sea coastal region of Egypt hosts a noticeable record of Pleistocene raised coral terraces, which provide valuable insights into past sea-level changes, tectonic activity, and paleoclimatic conditions. These raised coral structures are the remnants of ancient fringing reefs that developed during interglacial highstands and were subsequently uplifted or preserved above the current sea level. Along the western margin of the Red Sea, particularly in areas such as Gebel El-Zeit, Safaga, and Quseir, well-preserved Pleistocene reef terraces occur at various elevations.^40^ These terraces typically consist of three distinct reef units, which are widely correlated with Marine Isotope Stages (MIS) 5, 7, and 9-each representing interglacial periods when global sea levels were significantly higher than today. The coral assemblages within these units are dominated by reef-building taxa such as *Porites*, *Acropora*, and *Favites*, reflecting the warm, shallow marine conditions that prevailed during their formation.^41^ Geochronological studies using uranium-series dating have verified the ages of these terraces, particularly those of MIS 5e (around 125 000 years ago), which is the most extensively preserved unit.^42^ In the Gebel El-Zeit area, just the middle and lower reef units are observed, with the upper unit often absent—likely due to erosion, non-deposition, or paleotopographic controls. These terraces are typically found between 5 to 30 meters above current sea level, indicating either relative sea-level changes or tectonic uplift. Structurally, the region is influenced by the tectonic dynamics of the Red Sea rift system, which has played a role in the differential uplift of these coral terraces. While some sectors appear tectonically stable, other areas exhibit signs of long-term uplift. For example, uplifted terraces in the Gulf of Suez reflect both eustatic and tectonic influences on their elevation and preservation. Pleistocene raised reefs in Egypt not only serve as important archives of Quaternary sea-level history but also as analogs for modern reef development under changing climatic and oceanographic conditions.^43^

## 3. Materials and methods

### 3.1. The utilized materials and chemicals

The utilized HC samples were collected from raised coral deposits at the shoreline in the Gebel El-Zeit district of the northern Red Sea in Egypt to represent the coral limestone waste matrix used for CAC preparation. Samples were taken from the outer surface layer (10 cm) of the skeleton and from multiple points to obtain a representative composite sample. Hydrated aluminum nitrate Al(NO_3_)_3_·9H_2_O, with a purity of 99%, and nitric acid with a purity of 69% were provided from LOBA Chemical Company, Mumbai, India. The solution pH values were modified using either HCl (0.01 mol L^−1^, purity: 37%) or NH_4_OH (0.01 mol L^−1^, purity: 99%), which were delivered from LOBA Chemical Company, Mumbai, India.

### 3.2. Preparation of CAC adsorbent

The investigated HC sample was crushed to yield a fracture, which was ultrasonically washed with distilled water before being allowed to dry at 25 °C/48 h followed by drying at 70 °C/24 h. The dried fragment (approximately 100 g) of HC biowaste was received a further grinding process *via* a high-speed ball mill (Retch, Germany) to obtain particles minor than <63 µm, with their chemical composition listed in Table 1.

**Table 1:** Chemical constituents of the HC sample used in the preparation of CAC adsorbent

Oxides	Na_2_O	MgO	Al_2_O_3_	SiO_2_	P_2_O_5_	SO_3_	K_2_O	CaO	SrO	Cl	Br	LOI
Weight (%)	0.445	0.10	0.043	0.183	0.03	0.15	0.03	55.32	0.205	0.066	0.047	43.28

**Fig. 1:**
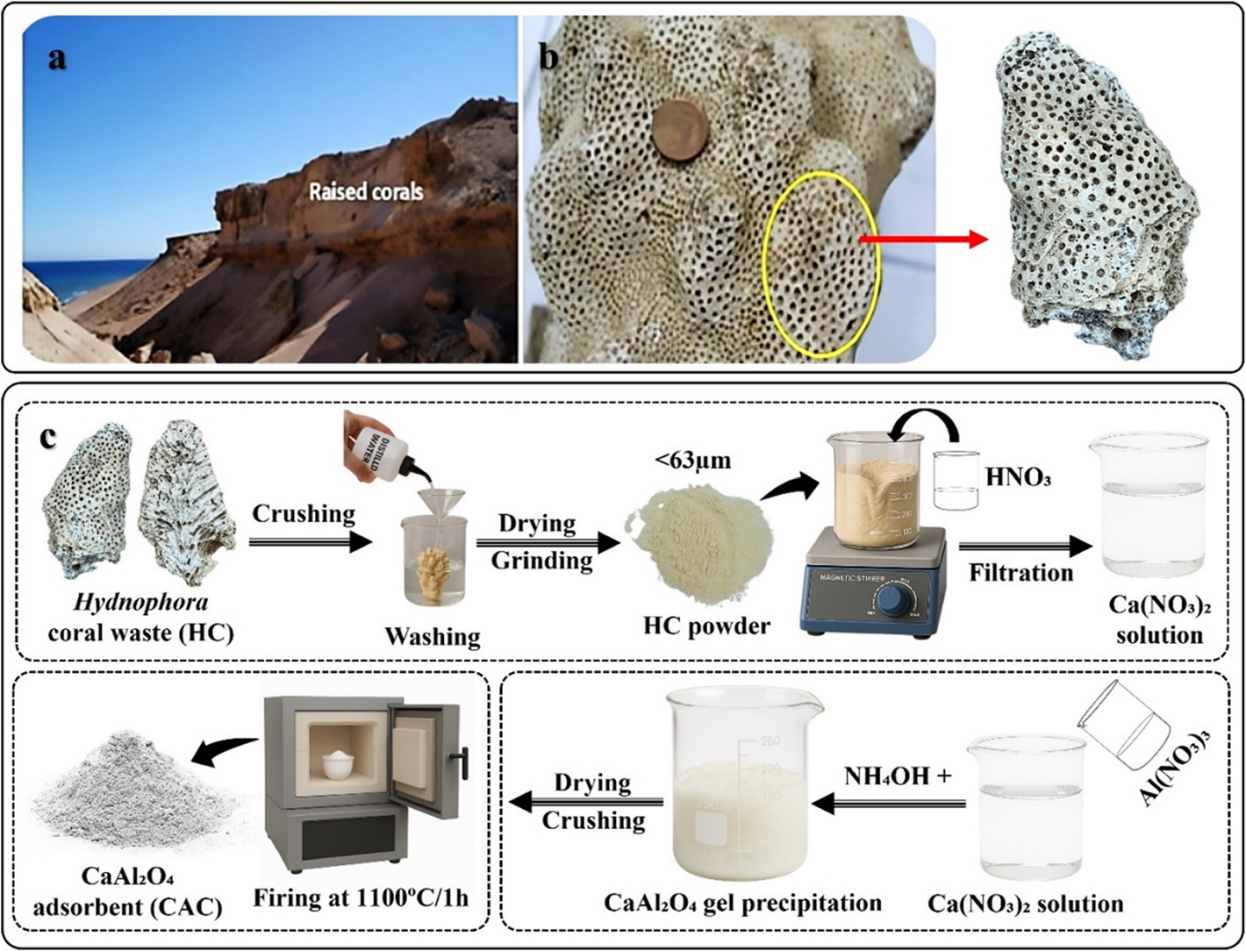
Field photo for the raised coral reefs along the Red Sea coast, Gabal El-Zeit (a) the utilized *Hydnophora* sp. (b), and the main steps used in the preparation of CAC from this limestone coral (c).

The synthesis of CaAl_2_O_4_, was achieved by converting the limestone HC waste (nearly 98.52% of CaCO_3_ calcite^35^ into calcium nitrate [Ca(NO_3_)_2_] *via* a solution of nitric acid (HNO_3_), under a constant stirring. For the fabrication of CaAl_2_O_4_, a stoichiometric value of aluminum nitrate solution was progressively added to the Ca(NO_3_)_2_ solution with stirring at 25 °C. For attaining the precipitation process, the pH value was adapted by adding ammonia solution until it reached the value of 9.5. Subsequently, the subsequent precipitated was dehydrated in a dryer at 70 °C for 48 h and then, crushed in an agate mortar. The resultant powder was treated at a temperature of 1100 °C/1 h. In brief, the developed CAC adsorbent (see Fig. 1c) was prepared, according to the following equations:

1 HC-CaCO_3_ (s) + 2HNO_3_ (aq) → Ca(NO_3_)_2_ (aq) + CO_2_ (g) + H_2_O

2 Ca(NO_3_)_2_ (aq) + Al(NO_3_)_3_ (aq) → CaAl_2_O_4_ (aq)

3 CaAl_2_O_4_ (aq) + NH_4_OH (aq) → CaAl_2_O_4_ (gel)

After formation, the CaAl_2_O_4_ gel was dried and heat-treated (1100 °C/1 h) to yield crystalline CAC, according to the next equation:

4 CaAl_2_O_4_ (gel form) → CaAl_2_O_4_ powder (*i.e.*, the CAC adsorbent)

### 3.3. Characterization of CAC adsorbent

The well-known phases were recognized though X-ray diffraction analysis (XRD, X-ray diffractometer model BRUKER Axs, D8 ADVANCE, Germany) with Cu Kα radiation (*λ* = 1.5406 Å), operating at 40 kV and 30 mA, a scan speed of 5° min^−1^, the diffraction were detected at angles between 5° and 60°. The active functional sites of the CAC adsorbent were examined by Fourier-transform infrared spectroscopy (FTIR) using a JASCO FT/IR-4600 spectrometer (JASCO Corporation, Hachioji-shi, Tokyo, Japan). The microstructure and morphological characteristics of the as-synthesized CAC adsorbent were examined using transmission electron microscopy (TEM) and scanning electron microscopy (SEM) equipped with energy-dispersive X-ray spectroscopy (EDX) techniques (JEOL, Tokyo, Japan).

### 3.4. Adsorption experiments

First, 1000 mg L^−1^ of Cr(vi) was organized into a stock solution, which was then diluted to reach the required concentrations. Important factors that control the adsorption process were described in the current work, including CAC mass, temperature, contact time, and solution pH value. Every experiment involving the adsorption of Cr(vi) by CAC was conducted on three separate cases and the outcomes were averaged. Furthermore, kinetics, isotherms, and thermodynamics parameters were considered to clarify the adsorption mechanism.

### 3.5. Cr(vi) adsorption kinetics

The adsorption conduct of Cr(vi) was evaluated at 25 °C under a constant agitation (150 rpm) across time intervals between 5 to 360 min. Following each interaction time, centrifugation was carried out to isolate the CAC phase from the liquid one. The experiment maintained fixed conditions: pH 2.0, 50 mg of CAC adsorbent, and 50 mL of Cr(vi) solution. A UV-Vis spectrophotometer was used to measure the lasting concentration of Cr(vi) following the centrifugation process, and the adsorbed amount of Cr(vi) was computed using the subsequent formula at each time interval:^27^5
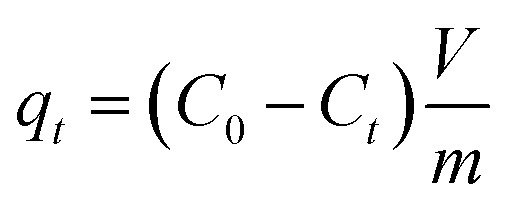
where *C*_0_ is the original Cr(vi) concentration and *C*_*t*_ is the is the concentration remaining at time (*t*). *V* is the solution volume (L) and *m* is the CAC mass (g).

To investigate the kinetics and rate-determining mechanisms of Cr(vi)–CAC interface, the corresponding calculations were fitted to pseudo-first-order (PFO),^44^ pseudo-second-order (PSO),^45^ and intra-particle diffusion (IPD).^46^ The mathematical expressions of these models are given below:

6 *q*_*t*_ = *q*_e_ e^−*k*_1_*t*^ (PFO)

7
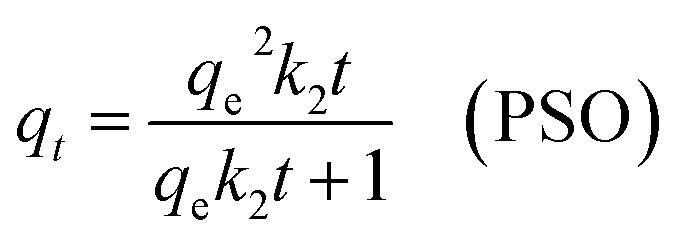

8 *q*_*t*_ = *k*_p_*t*^1/2^ + *C* (IPD)

where the adsorption rate constants of the PFO and the PSO are donated by *k*_1_ (min^−1^) and *k*_2_ (g mg^−1^ min^−1^), respectively. The rate constant and the intercept value are contributed to the *k*_p_ [(mg g^−1^)(min^−1^)^0.5^)] and *C* (mg g^−1^) of the IPD equation, respectively.

### 3.6. Application of classical models for Cr(vi) uptake data

The adsorption isotherms associated with the Cr(vi)–CAC interface were conducted using Cr(vi) solutions with initial concentrations varying between 10 and 80 mg L^−1^. The other constant parameters were as follows: pH (2.0), CAC dosage (25 mg), solution volume (50 mL), and three temperatures (25, 40, and 55 °C). Based on the residual Cr(vi) concentration *C*_e_ (mg L^−1^) in solution, the equilibrium adsorption capacities (*q*_e_ (mg g^−1^)) were then determined through the following relation.9
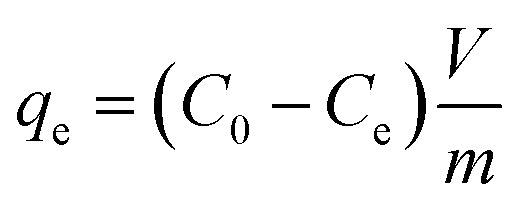


Non-linear formulations of three widely used isotherm models were used to analyze the Cr(vi)–CAC adsorption data. The corresponding equations of the applied common models (*i.e.*, the Langmuir,^47^ Freundlich,^48^ and Liu^49^ equations), as provided by the subsequent mathematic relations:10
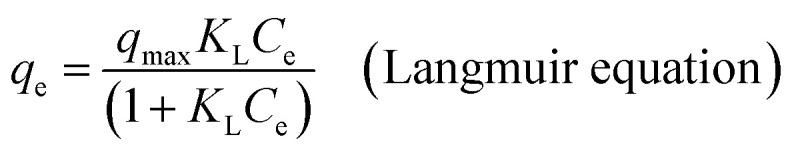

11 *q*_e_ = *K*_F_*C*_e_^1/*n*^ (Freundlich equation)

12
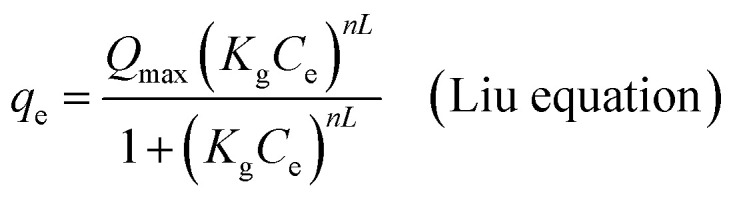
where *q*_max_ (mg g^−1^) and *K*_L_ (L mg^−1^) characterize the supreme removed amount of Cr(vi) and the Langmuir constant and Liu, respectively. *k*_g_ is the Liu equilibrium constant and *n*_L_ is the Liu exponent, while the *K*_F_ ((mg g^−1^)(mg L^−1^)^−1/*n*^)) and *n* are the constants of the Freundlich model that are associated with the adsorption capacities and intensities, respectively. Furthermore, though the determination coefficient (*R*^2^) and Chi-square (*χ*^2^) values, the best-fitting model was found; the higher *R*^2^ and the lower *χ*^2^ values indicate the appropriate adsorption model:^19,29^13
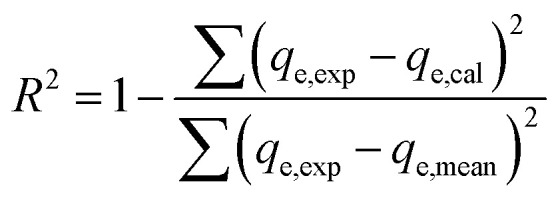
14
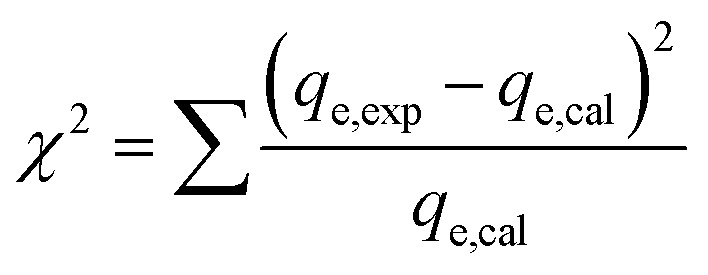
where *q*_e,exp_ (mg g^−1^) and *q*_e,cal_ (mg g^−1^) are the experimental and the hypothetical values of Cr(vi) adsorption capacities, respectively.

### 3.7. Application of advanced models for Cr(vi) adsorption data

Since the simple Freundlich, Liu, and Langmuir models have no physical significance, it is generally necessary to make clear that these equations are insufficient to provide the physico-chemical factors, such as steric and energetic characteristics, that significantly influenced the control of the adsorption systems. In this study, various statistical models were applied to gain deeper insight into the adsorption behavior and to assess the efficiency of the CAC adsorbent in capturing Cr(vi) ions. To have a comprehensive understanding of the CAC-Cr(vi) mechanism, monolayer (ML), double-layer (DL), and multilayer (MuL) statistical models were taken into attention.

#### 3.7.1. Statistical ML model

According to the ML, the Cr(vi) uptake *via* CAC was linked to a sole process, meaning that the ions that were removed might have formed a single layer as a result of the interface interaction between Cr(vi) and the CAC surface. The model's mathematical expression is given below.^19^15
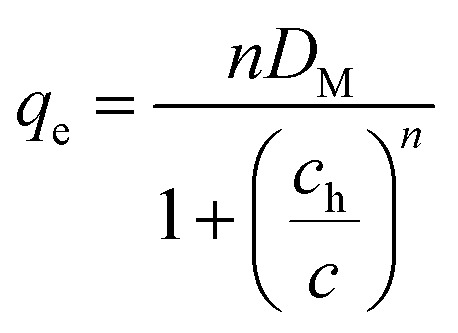
where *D*_M_ represents the density of CAC, *C*_h_ is the half-saturation concentration related to the generated adsorbate layer, and *n* is the number of Cr(vi) ions eliminated by the CAC active site.

#### 3.7.2. Statistical DL model

The DL model proposes the construction of two adsorbate layers of the confirmed Cr(vi) ions with two separate energies (*i.e.*, the first interface energy (Δ*E*_1_) corresponds to the first layer, while the second uptake energy (Δ*E*_2_) characterizes the interactions between the chromium ions). DL's mathematical expression is shown in earlier research.^29,35^16
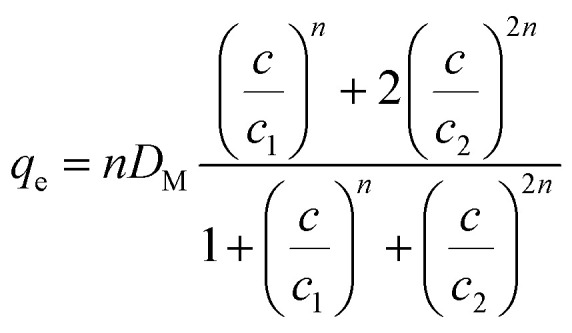
where the two layers that develop on the CAC surface are responsible for the identification of two concentrations at half-saturation (*C*_1_ and *C*_2_).

#### 3.7.3. Statistical MuL model

The existence of a given number of Cr(vi) layers that are governed by different energies (*i.e.*, Δ*E*_1_ and Δ*E*_2_) above the CAC surface is the key factor of the MuL suggestion.^19^ Consequently, a total of 1 + N_2_ layers can be formed on the CAC surface. The initial layer results from Cr(vi)–CAC surface interaction is characterized by an energy change (Δ*E*_1_), while the additional N_2_ layers are ascribed to adsorbate–adsorbate connections with a linked energy (Δ*E*_2_), where Δ*E*_1_ > Δ*E*_2_. The calculated number of Cr(vi) layers on CAC is equal to 1 + N_2_. The corresponding parameters for the MuL model were derived using the subsequent equations:^35^17
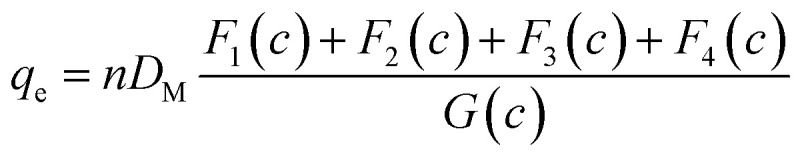
18
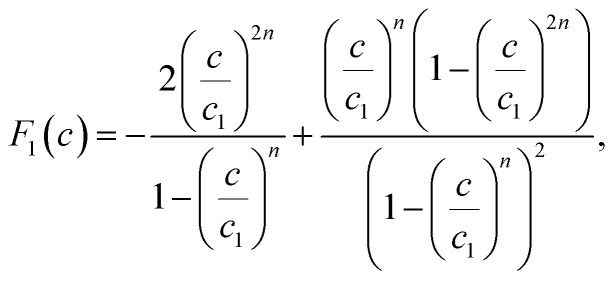
19
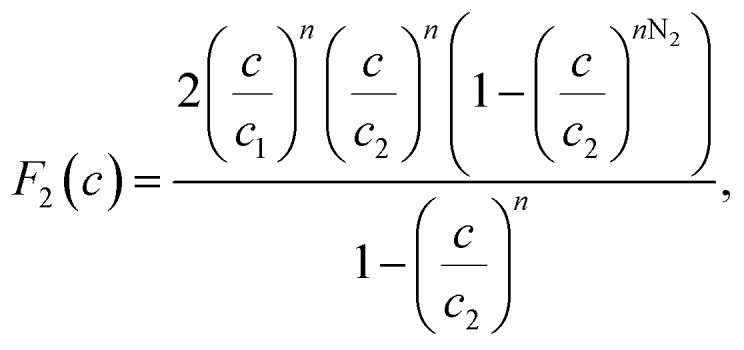
20
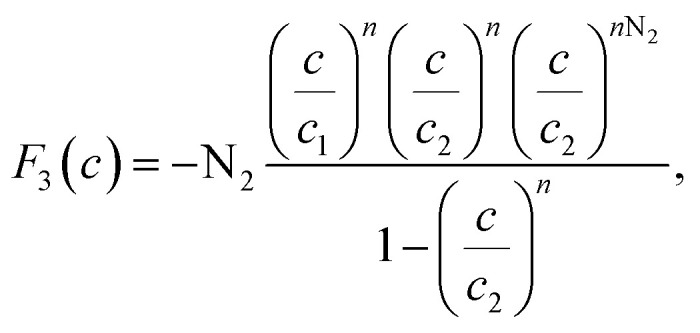
21
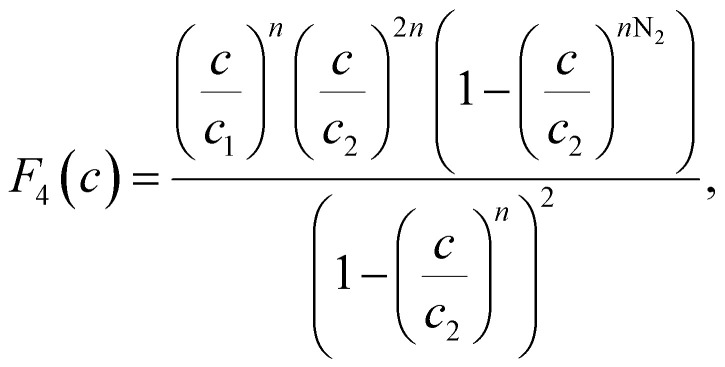
22
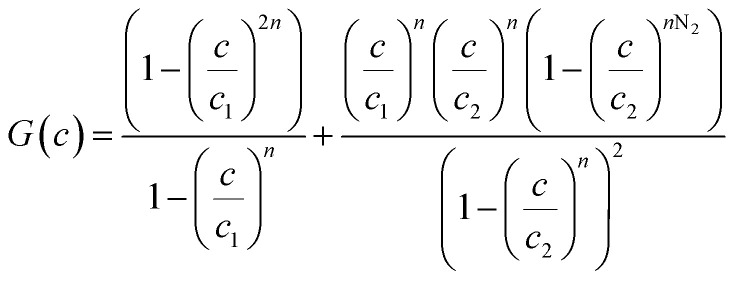


Overall, the MuL model can be linked to the following operating settings (S1–S5):^35^ S1: Both *n* and N_2_ are freely adjustable, producing a multilayer model. S2: *n* is variable while N_2_ is set to zero, signifying a monolayer model. S3: *n* is variable and N_2_ equals 1, corresponding to a double-layer model. S4: *n* is variable and N_2_ equals 2, indicating a triple-layer model. S5: *n* is fixed at unity and N_2_ is zero, conforming to the Langmuir model.

In addition to the values of *R*^2^, the root mean square error (RMSE) values were used to support the selection of the best advanced statistical model.^19^23
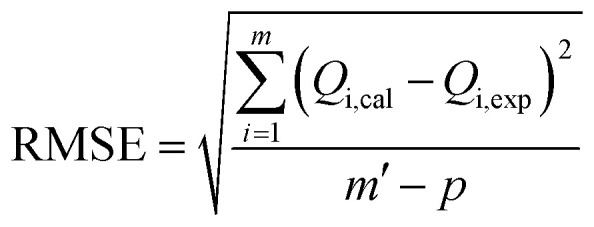
where *m*′ denotes the experimental data and *p* is the number of adjustable parameters.

### 3.8. Cr(vi) uptake thermodynamics

Under the suitable experimental conditions (*i.e.*, pH of 2.0, CAC dose of 200 mg, and shaking duration of 120 min), thermodynamic characteristics of Cr(vi) adsorption on the CAC were measured at temperatures between 25 and 55 °C. Gibbs free energy (Δ*G*^0^, kJ mol^−1^), enthalpy change (Δ*H*^0^, kJ mol^−1^), and entropy variation (Δ*S*^0^, kJ mol^−1^. K) were among the thermodynamic parameters used in this study. These parameters offer understandings into the viability, spontaneity, and nature (exothermic or endothermic) of the Cr(vi) adsorption mechanism.

24 Δ*G*^0^ = −*RT* ln(*K*_e_) = Δ*H*^0^ − *T*Δ*S*^0^

25
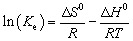
26
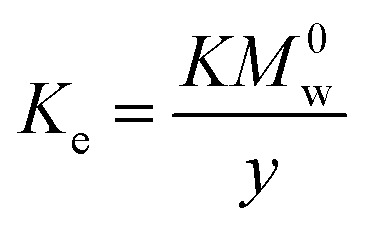
where *T* is the temperature (K), *R* is the universal gas constant (8.314 J mol K^−1^), *M*^0^_w_ is the molecular weight of the adsorbate (mg mol^−1^), *y* is the unit activity coefficient, *K*_e_ is the equilibrium constant, and *K* is the constant based on the best-fit parameter of the isothermal model (L mg^−1^).

### 3.9. Reusability study of CAC adsorbent

The reusability of the Cr(vi)–CAC adsorbent was investigated to evaluate the stability of this ceramic product for sustainable wastewater treatment applications. The Cr(vi)–CAC adsorbent was regenerated at 25 °C using 100 mL of 0.5 M NaOH as a desorbing agent. The suspension was agitated in a mechanical shaker (SHO-2D Shaker, Germany) at 150 rpm for 3 h to facilitate the desorption of Cr(vi) ions from the active sites of the CAC adsorbent. The adsorption–desorption procedure was carried out for five consecutive cycles, keeping the same operational parameters for each cycle to evaluate the reusability of CAC product. The CAC was dried at 70 °C for 12 hours and cleaned three times with distilled water at the finish of each desorption round. The Cr(vi) removal efficiency after each adsorption–desorption round was measured and compared to that of the as-prepared CAC material to evaluate the regeneration performance and structural stability of the ceramic adsorbent.

## 4. Results and discussions

### 4.1. CAC adsorbent characterization

**Fig. 2:**
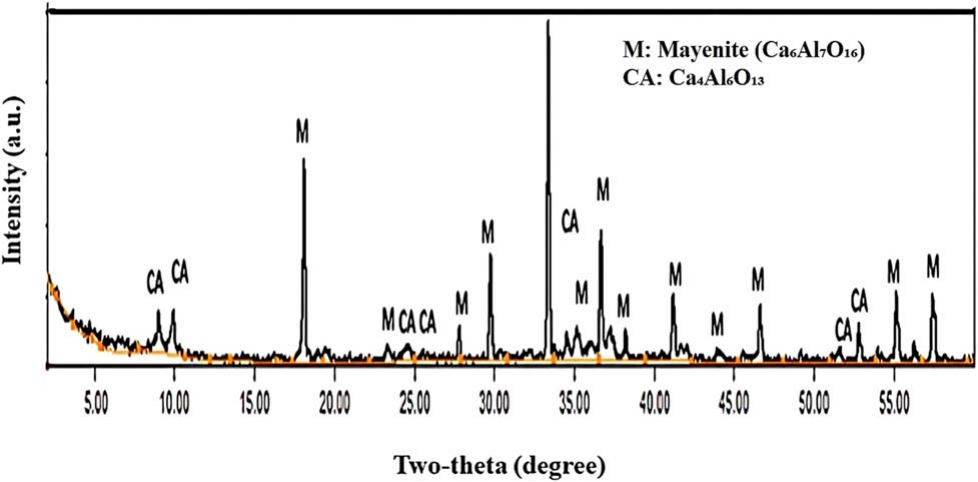
X-ray diffraction patterns of the as-synthesized CAC product.

Fig. 2 shows the XRD pattern of the formed CAC after sintering the HC sample at 1100 °C/1 h. This pattern shows the presence of two crystallization Ca and Al oxides with different crystal systems. Mayenite (Ca_12_Al_14_O_33_, cubic, ICDD # 96-901-1337) and Ca_4_Al_6_O_13_ (orthorhombic, ICDD # 96-900-2487) were the main detected phases (Fig. 2). We are able to observe that the “M” peaks (Mayenite) are noticeably more intense than the “CA” peaks at these sintering conditions. This implies that Ca_4_A_l6_O_13_ is a minor phase in the sample, while mayenite is the main crystalline phase. Both M and CA phases are highly crystalline, but the quantity of M phase was higher as compared to the CA one, as seen in Fig. 2. Furthermore, an amorphous component was also detected in the sample, as indicated a broad and low-intensity background signal, but this is negligible in comparison to the crystalline phases. This result was consistent with a previous study that found that crystalline mayenite and CA phases were the primary detected phases between 900 and 1100 °C, while amorphous or poorly crystalline Ca and Al-rich phases were observed at firing temperatures below 900 °C.^50^

**Fig. 3:**
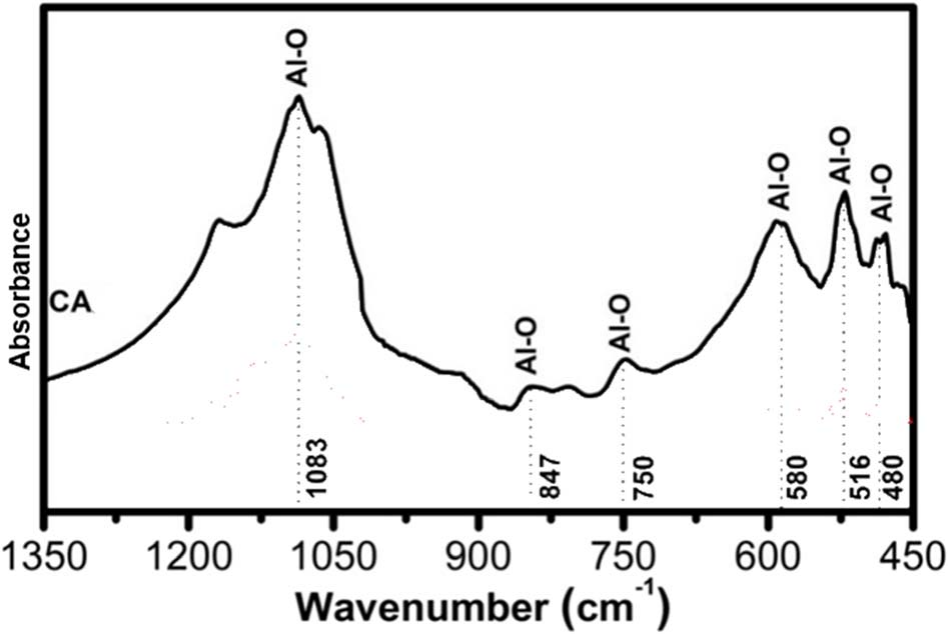
FTIR spectrum of the developed CAC adsorbent.

Fig. 3 shows the FTIR spectrum of the CAC sample sintered at 1100 °C for 1 h. The low-wavenumber bands at 580, 516, and 480 cm^−1^ are assigned to Al–O lattice vibrations mainly associated with AlO_6_ octahedral units,^25,26^ confirming the formation of the calcium aluminate framework.^50,51^ The bands at 750 and 847 cm^−1^ are attributed to Al–O–Al/Al–O vibrations within the C_12_A_7_ phase aluminate network (AlO_4_/AlO_6_ linkages).^50^ The strong band at 1083 cm^−1^ is also related to Al–O stretching in the aluminate (AlO_4_ tetrahedra) structure, further supporting the development of calcium aluminate phases after sintering.^52^

**Fig. 4:**
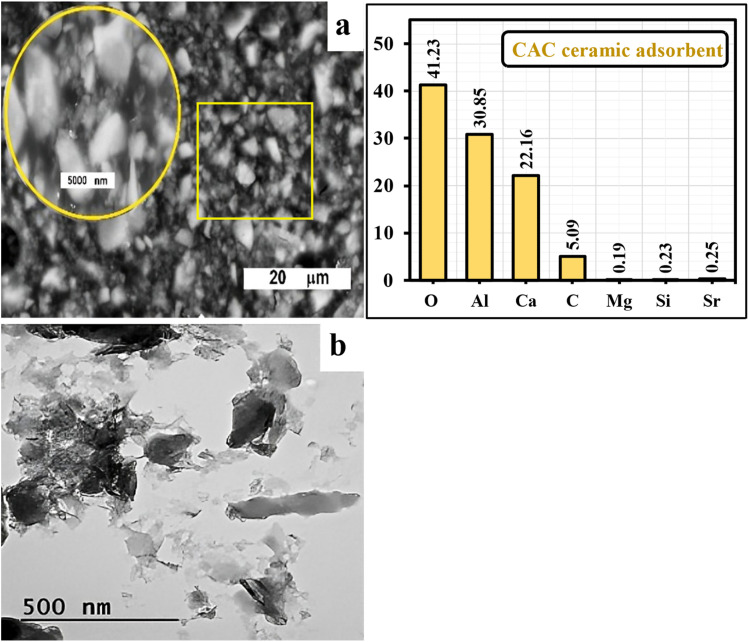
SEM (a) and TEM (b) images of the developed CAC adsorbent.

SEM result of the developed CAC biosorbent, at two magnifications, is shown in Fig. 4a. The low-magnification image (20 µm scale bar) displays a compact microstructure that is composed of angular and unevenly shaped grains with wide-ranging particle sizes. On the other hand, the higher magnification region (identified within the yellow circle) reveals the finer textural details of the CAC adsorbent matrix, showing closely packed grains with smoother surfaces and dissimilar grain boundaries. The microstructure indicated well-developed crystallinity of calcium aluminate phases formed at high temperature. The EDX spectrum of CAC is dominated by the framework elements O (41.23 wt%), Al (30.85 wt%), and Ca (22.16 wt%), which is consistent with the formation of calcium aluminate phases. A small C signal (5.09 wt%) is typically attributed to adventitious carbon and/or sample coating during SEM analysis, while only trace contributions of Mg (0.19 wt%), Si (0.23 wt%), and Sr (0.25 wt%) are detected, likely inherited from minor impurities in the biogenic precursor. Importantly, no Cr signal is observed before adsorption, providing a clear baseline for confirming chromium loading after treatment.

Agglomerated particles with a range of contrasts and morphology, from amorphous to polycrystalline, are visible in the TEM image (Fig. 4b). The image is dominated by irregular flakes and platelet-like features. Transparent areas around darker cores in a number of spots, suggesting porous or less dense materials that may be the result of nano-porosity or the existence of an amorphous matrix. A mixed-phase system is suggested by contrast and granularity variation. Crystalline calcium aluminate granules are probably found in dark areas. Areas that are lighter could be unreacted alumina/calcium oxide or an amorphous Ca–Al–O matrix. The estimated gran sizes range from 80 to 150 nm in the figure's center, while the flake-like particles range from 100 to 200 nm.

### 4.2. Effect of pH

**Fig. 5:**
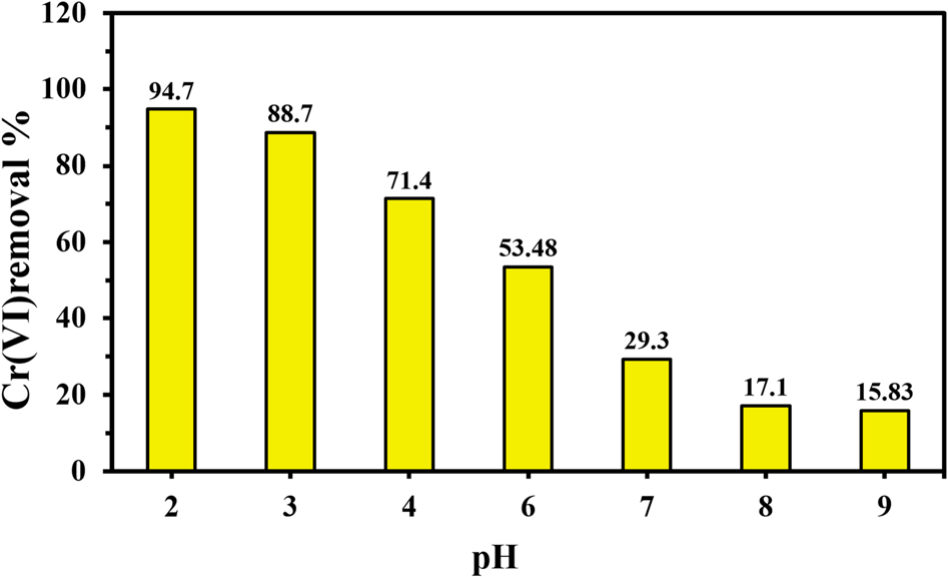
Effect of solution pH on Cr(vi) uptake by CAC (25 mg), at 50 mg L^−1^ Cr(vi), 2 h contact time, and 25 °C.

A pH range of 2.0 to 9.0 was selected to assess the CAC adsorbent's optimal performance in removing Cr(vi) ions. The pH experiment was carried out using 25 mg of CAC, a Cr(vi) concentration of 50 mg L^−1^, a 2 hour interaction time, and a 25 °C adsorption temperature. The results pertaining to the pH influence on the removal system are shown in Fig. 5. At pHs 2.0 (94.52%) and 3.0 (88.76), the maximum absorption percentages of Cr(vi) were presented; when the pH level increased above 3.0, the Cr(vi) uptake percentage declined. The CAC adsorbent's functional groups were protonated at pH values of 2.0 and 3.0 due to the high concentration of H^+^ in the solution.^53^ The main form of hexavalent chromium, HCrO_4_^−^, and the protonated active sites of CAC interact strongly to generate the greatest chromate uptake percentages (Fig. 5). Furthermore, the reduction of HCrO_4_^−^ at these low pH values could result in a substitution reaction between the positively charged functional structures of CAC and the reduced Cr^3+^ ions. Thus, the percentage of Cr(vi) removal was increased by both electrostatic and substitution reactions.^53^ The deprotonation of CAC's active sites caused the Cr(vi) absorption percentages to decline, showing values of 71.4% and 53.48%, respectively, as the pH enhanced to 4.0 and 6.0. The presence of hydroxyl ions was thought to be the primary cause of the decline in the capture of Cr(vi), especially at solution pH values higher than 7.0. This behavior could be related to the electrostatic repulsion force between the CrO_4_^−2^ and the negative adsorption sites of the CAC and thus, all Cr(vi) studies were carried out at pH 2.0.

### 4.3. Effect of CAC mass

**Fig. 6:**
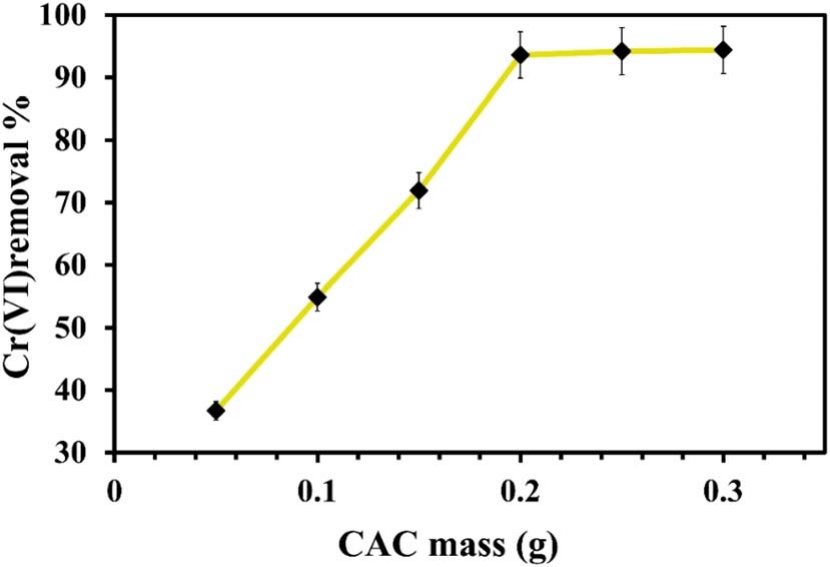
Effect of CAC dose on the removal of Cr(vi) ions.

To assess the impact of dose on the percentage of Cr(vi) elimination, several masses of the CAC, ranging from 50 to 300 mg, were utilized. After mixing 25 mL of Cr(vi) with 150 mg L^−1^ of concentration, each mass was shaken for two hours at 120 rpm. Fig. 6 shows how the adsorbent mass affects the percentage of Cr(vi) absorbed from the aqueous solution. As shown in Fig. 6, the removed percentages of Cr(vi) rose as the CAC dose was raised from 0.05 to 0.2 g. This may be explained by the CAC's increased surface area overall and the presence of more active sites accessible for the elimination of Cr(vi) ions.^54^ Due to the saturation of CAC active sites at this concentration of the measured adsorbate, the increment of CAC masses did not clearly increase the chromium adsorption percentages over 200 mg of CAC dosage. Thus, 200 mg of CAC was suggested to be the optimal dosage required to extract chromium ions from aqueous solutions under these experimental parameters.

### 4.4. Kinetics study and Cr(vi) diffusion mechanism

**Fig. 7:**
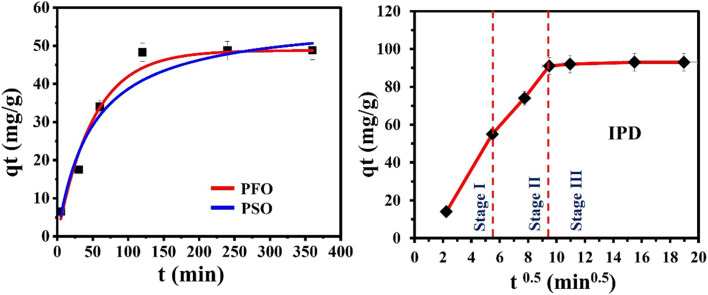
Kinetics study of Cr(vi) removal on CAC, including the pseudo-first-order (PFO), the pseudo-second-order (PSO), and intraparticle diffusion (IPD) models.

The non-linear methods of the PFO and PSO kinetics resulted from the (*q*_*t*_) against (*t*) relationships (Fig. 7), and the subsequent calculations are presented in Table 2. Based on the values of determination coefficients (Table 2), the PFO kinetic model (*R*^2^ = 0.9657) provided a better fit for Cr(vi) adsorption as compared to the PSO equation (*R*^2^ = 0.9426). Moreover, the similarity between the calculated *q*_e_ = 50.89 mg g^−1^ and the experimental *q*_e_ = 48.37 mg g^−1^ of the PFO model, at all initial Cr(vi) concentrations, supporting the capability of the PFO equation in fitting the Cr(vi)–CAC adsorption kinetics. Therefore, the full Cr(vi) uptake rate using the developed adsorbent was largely governed by a physical interaction between Cr(vi) ions and CAC's active sites.^35^

**Table 2:** Kinetic parameters of Cr(vi) adsorption on CAC adsorbent

Kinetic models	Parameters		*R* ^2^
PFO	*q*_e_ (mg g^−1^)	*k*_1_ (min^−1^)	0.9657
	50.888	0.0199	
PSO	*q*_e_ (mg g^−1^)	*k*_2_ (g mg^−1^ min^−1^)	0.9426
	57.527	3.566	
IPD	*k*_p_ [(mg g^−1^) (min^−1^)^0.5^)]	*C* (mg g^−1^)	0.8072
	2.612	7.45	

The behavior of the Cr(vi) adsorption process onto the CAC cannot be explained by either the PFO or the PSO equations. In order to assess the rate directing the diffusion of Cr(vi) ions, the intra-particle diffusion (IPD) model was considered.^46^ A linear plot of *q*_*t*_ against *t*^0.5^ with an intercept (*c*) value of zero suggests that the IPD is the only rate controlling step, a multi-linear plot with intercept (*C*, mg g^−1^) ≠ zero suggests that other mechanisms, like film diffusion, are involved in the uptake process.^35^ The IPD was separated into distinct linear periods over the entire time range (5–360 min), as shown in Fig. 7. These parts characterize the film diffusion, the pore-diffusion, and equilibrium stages. Consequently, the Cr(vi) capture mechanism on the CAC adsorbent was primarily controlled by a mixture of film diffusion and IPD mechanisms.^53^

### 4.5. Simple equilibrium models

**Fig. 8:**
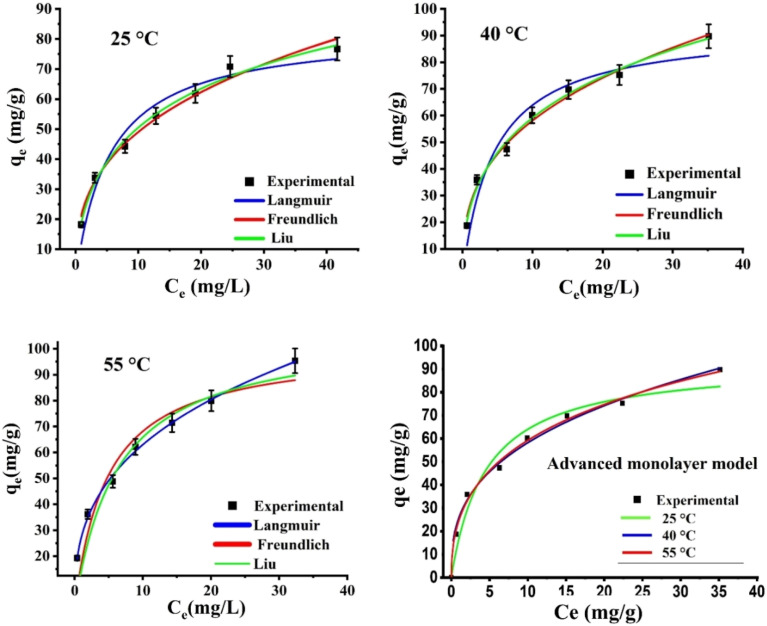
Calculations of modeling Cr(vi) adsorption on CAC using Liu, Freundlich, Langmuir, and monolayer equilibrium models at different temperatures.

The measurements of the Liu, Langmuir, and Freundlich equilibrium models that were used in this study were examined at 25, 40, and 55 °C. As shown in Fig. 8, the non-linear technique was used to analyze each adsorption model's equation. Furthermore, Table 3 lists the corresponding parameters for each isotherm model at all solution temperatures. According to the Langmuir model, each active site on the CAC surface has the same energy and forms a single layer. The Freundlich isotherm model, on the other side, suggests that the adsorption process occurs in several layers on the surface of the adsorbent without reaching saturation, with varying energy levels at each active site. Furthermore, the classical Liu's model combines aspects of the Freundlich and Langmuir models.^49^ The Liu equation categorizes the saturation of the adsorbent, while permitting several adsorption layers and active sites with different energy levels. The adjusted *R*^2^ and *χ*^2^ values were used to assess how well the models represented the experimentally obtained data. The results demonstrated that the Liu isotherm model was the most appropriate for explaining the experimental data since it yielded the greatest adjusted *R*^2^ (>0.99 at every temperature) and the lowest *χ*^2^ values. The Liu model's *q*_max_ increased from 148.65 to 304.62 mg g^−1^ within the 25–55 °C solution temperature range, indicating an endothermic interface between the CAC and the attached Cr(vi) ions. Additionally, a possible absorption system was indicated by the heterogeneity parameter (1/*n*_F_ < unity) determined using the Freundlich equation, and the linked Cr(vi) ions are not actually removed from the CAC surface.^29,35^

Equilibrium parameters of dissimilar adsorption models for Cr(vi) uptake by CAC adsorbent

**Table:** 

Isotherm model	Parameters				
	*T* (°C)	*q*_max_ (mg g^−1^)	*K*_L_ (L mg^−1^)	*R* ^2^	*χ* ^2^
Langmuir	25	82.93	0.1837	0.9543	13.68
	40	93.16	0.2182	0.9377	14.52
	55	101.01	0.2701	0.9230	16.50

**Table:** 

Isotherm model	Parameters				
	*T* (°C)	*K*_F_ ((mg g^−1^)(mg L^−1^)^−1/*n*^)	1/*nF*	*R* ^2^	*χ* ^2^
Freundlich	25	22.15	0.345	0.9841	6.28
	40	25.91	0.350	0.9897	7.34
	55	27.83	0.354	0.9971	2.17

**Table:** 

Isotherm model	Parameters					
	*T* (°C)	*q*_max_ (mg g^−1^)	*K*_g_	1/*nF*	*R* ^2^	*n*_L_
Liu	25	148.65	0.0288	0.9911	3.11	0.553
	40	269.66	0.0311	0.9917	2.98	0.446
	55	304.62	0.0512	0.9926	2.41	0.325

### 4.6. Advanced modeling analysis

The fitting results demonstrated a strong agreement between the Cr(vi) experimental data and the advanced ML model, with *R*^2^ values exceeding 0.98, as shown in Fig. 8. Accordingly, the steric and energetic factors obtained from the ML model were utilized to interpret the interaction mechanism between the Cr(vi) ions and CAC active sites at all investigated adsorption temperatures.

#### 4.6.1. Steric parameters

The steric parameter *n* provides insight into the behavior and surface geometry influencing the adsorption performance of Cr(vi) onto the as-synthesized CAC adsorbent. This parameter reflects three distinct adsorption scenarios for Cr(vi), as summarized in Table 4.^19^

**Table 4:** The cases of *n* parameter and the corresponding geometry and mechanism

Case number	Explanation
First, *n* < 0.5	Two or more CAC active sites can adsorb Cr(vi), enabling a multi-docking removal process with a horizontal orientation
Second, *n* between (0.5 and 1)	A mixed geometry with parallel and non-parallel orientations is detected
Third, *n* > 1	The functional groups on CAC can remove one or more Cr ions with a vertical's orientation and a multi-interaction mechanism

**Fig. 9:**
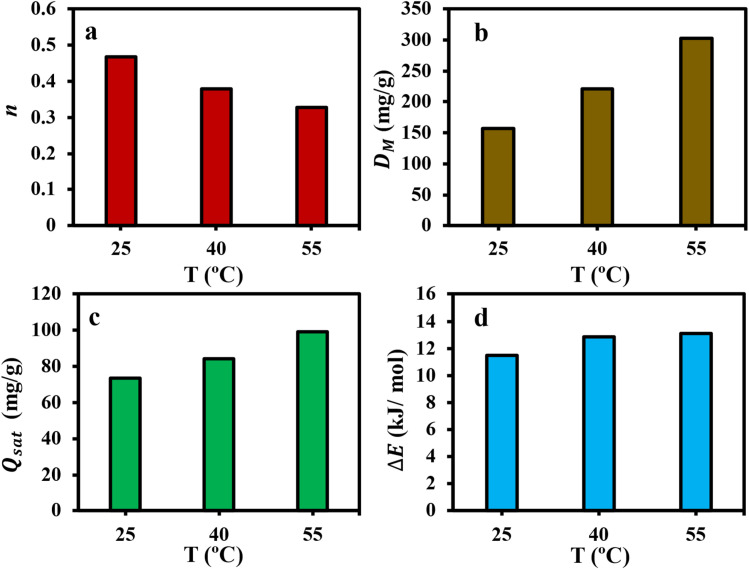
Physico-chemical parameters associated with Cr(vi) uptake at different temperatures, including steric (a–c) and energetic (d) parameters.

Fig. 9 shows the dissimilarity of *n* values with adsorption temperature. All values remained below 0.5, indicating that the *n* factor corresponds to the first adsorption behavior case of Table 4. This trend suggested that Cr(vi) adsorption on the CAC surface occurred in a horizontal orientation, involving a multi-docking interaction mechanism. Additionally, changing temperature had a negligeable effect on the adsorption geometry and removal mechanism of Cr(vi) ions.

Fig. 9 illustrates the behavior of the steric *D*_M_ parameter for Cr(vi) removal by the CAC adsorbent over an extended temperature range (25–55 °C). As the temperature increased from 25 °C to 55 °C, the *D*_M_ value rose significantly from 157.28 mg g^−1^ to 302.35 mg g^−1^. This increase suggests that higher temperatures either revealed or generated additional effective adsorption sites for Cr(vi) capture. At elevated temperatures, the Cr(vi)–CAC interface appears to allow the obtainability of more energetic adsorption sites, leading to a greater number of the occupied active sites inside the CAC adsorbent. Moreover, the rise in *D*_M_ with temperature confirmed that the adsorption process is an endothermic in nature. Notably, the *D*_M_ and *n* parameters exhibited opposite trends with increasing temperature.

The steric parameter *Q*_sat_ = *n* × *D*_M_ is used to calculate the capacity of the CAC adsorbent to remove Cr(vi) ions. As shown in Fig. 9, the increase in *Q*_sat_ values with rising temperature indicated an endothermic interaction between the CAC and chromium ions. Both *Q*_sat_ and *D*_M_ parameters followed similar trends with temperature variation, suggesting that the *D*_M_ parameter played a key role in determining the adsorption efficiency of the CAC adsorbent. Overall, the interaction temperature was found to positively influence the adsorption capacity, likely by enhancing the mobility of Cr(vi) ions in solutions. This conduct resulted in increasing the *D*_M_ and *Q*_sat_ parameters, supporting the endothermic nature of the adsorption system.

#### 4.6.2. Energetic parameters explanation

**Fig. 10:**
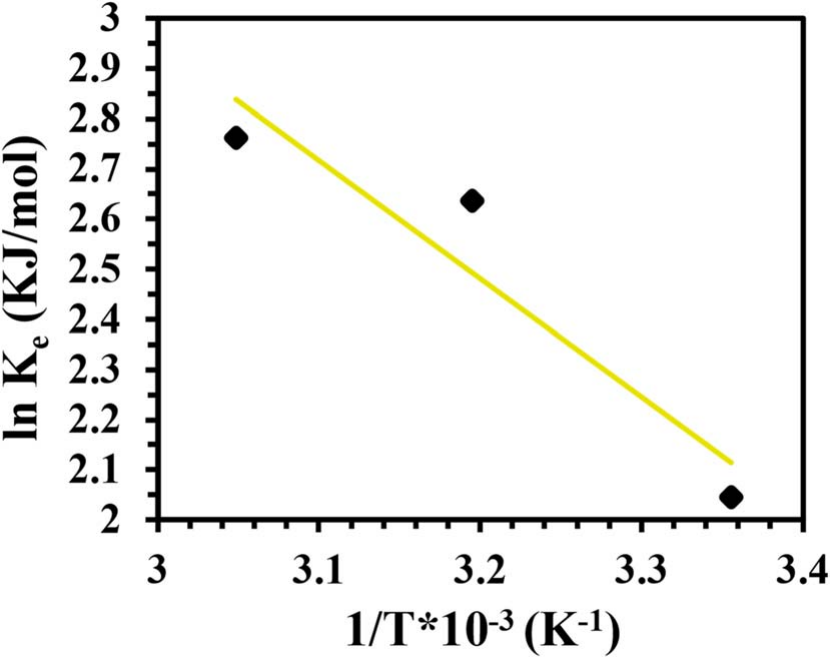
Thermodynamic functions associated with Cr(vi) uptake by CAC adsorbent.

Fig. 10 presents the adsorption energy values (Δ*E*) of Cr(vi) at different temperatures. The consistently positive Δ*E* across all tested temperatures confirmed the endothermic nature of the interaction between Cr(vi) ions and the CAC adsorbent. Moreover, the adsorption energies remained below 15 kJ mol^−1^, indicating that the process was primarily driven by physical forces such as electrostatic interactions and pore filling. Overall, the behavior of both Δ*E* and *D*_M_ parameters at each temperature reflected their dominant role in governing the Cr(vi) adsorption mechanism, as revealed by the steric and energetic analyses.

### 4.7. Thermodynamics study

Thermodynamic parameters associated with Cr(vi) adsorption on CAC were computed using the linear technique (Fig. 9); the outcomes are shown in Table 5. These thermodynamics functions, which included entropy (Δ*S*), Gibbs free energy (Δ*G*), and enthalpy (Δ*H*), were calculated by doing a regression analysis of the related data. Clearly, the value of *K*_e_ improved as the solution's temperature increased, indicating that the Cr(vi) adsorption capacity increased at high temperatures (*i.e.*, the absorption system of Cr(vi) was of endothermic sort).^35^ Furthermore, the endothermic character of the interaction between CAC and Cr(vi) was validated by the positive Δ*H* value.^55^ At every solution temperature, the Δ*G* values were negative, indicating that the Cr(vi) adsorption system was spontaneous.^35^ Additionally, the computed Δ*H* value was less than 20 kJ mol^−1^, demonstrating that the uptake of Cr(vi) is physical in nature.^55^ Additionally, when the temperature increased, the Δ*G* value decreased (Table 5), signifying that high temperatures were conducive to Cr(vi) adsorption.^55^ Furthermore, the adsorption of Cr(vi) on CAC is a spontaneous physical adsorption method, as specified by the negative Δ*G* in the −20 to 0 kJ mol^−1^ region.^56^ The positive Δ*S* value designated the adsorbent's affinity for Cr(vi) and the increasing unpredictability at the solid–liquid interface through the removal system.^35,56^

**Table 5:** Thermodynamics factors related to Cr(vi) uptake using CAC adsorbent

*T* (K)	*K*_e_ (kJ mol^−1^)	Δ*H* (kJ mol^−1^)	Δ*S* (kJ mol^−1^ K^−1^)	Δ*G* (kJ mol^−1^)
298	7.729	19.601	83.34	−5.064
313	13.988			−6.861
328	15.841			−7.528

### 4.8. Regeneration study of CAC adsorbent

**Fig. 11:**
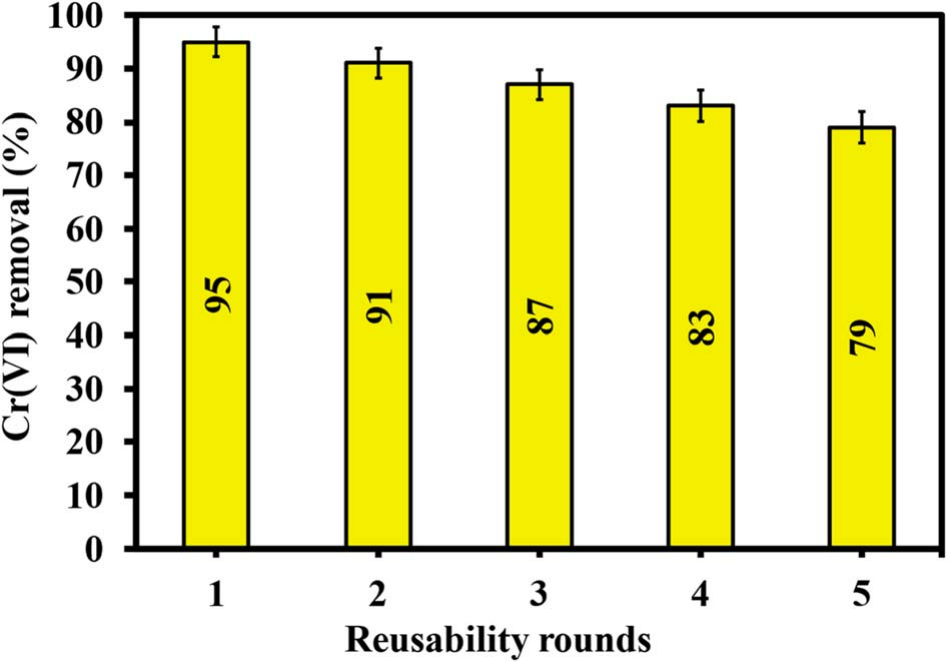
Reusability of CAC after five adsorption/desorption cycles.

The CAC adsorbent offered good regeneration capacity following the first, second, third, fourth, and fifth adsorption/desorption cycles (Fig. 11), yielding values of 95%, 91%, 87%, 83%, and 79% of Cr(vi) absorption, respectively. A possible clarification for the reduced performance of CAC after adsorption–desorption cycles is the mechanical weakening of the microporous matrix, as well as the potential trapping of Cr(vi) ions within the small pores of the developed ceramic sample. Therefore, the adsorption ability of CAC ceramic was not significantly reduced even after being recycled multiple times for the effective removal of Cr(vi) ions. As a result, CAC was found to be a promising ceramic-based adsorbent that effectively removes Cr(vi) from water. This ceramic material may find application in the industrial sector. Overall, CAC adsorbent can be used to treat wastewater that contains hexavalent chromium ions in a more economical and environmentally friendly approach.

### 4.9. SEM-EDX analysis of the spent CAC adsorbent

**Fig. 12:**
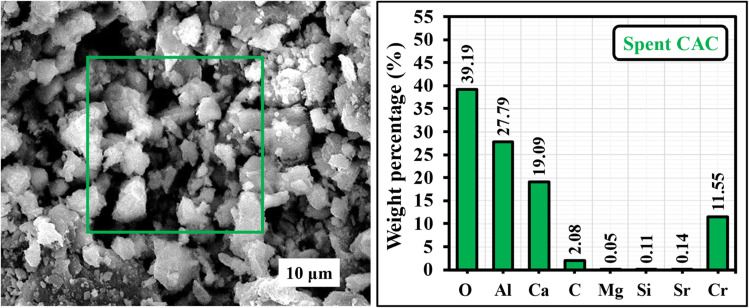
SEM-EDX micrograph of the spent CAC adsorbent, showing the post-regeneration surface morphology and elemental analysis.

After five adsorption–desorption cycles, the SEM micrograph reveals a rough, granular, and strongly agglomerated CAC surface decorated with fine bright particulates, suggesting surface coverage by adsorbed species after reuse. The EDX results clearly verify chromium retention on the spent material, as indicated by a pronounced Cr contribution (13.55 wt%), while the matrix elements remain predominant (O 39.19 wt%, Al 27.79 wt%, Ca 19.09 wt%), confirming that the calcium aluminate framework is largely preserved. Trace Mg, Si, and Sr signals are attributed to minor impurities originating from the biogenic precursor (Fig. 12).

### 4.10. Comparison study

Under identical conditions (25 °C; pH 2–3), CAC exhibits a higher adsorption capacity (*Q*_max_) than many benchmark adsorbents summarized in Table 6, including modified activated carbons, magnetic biochar, activated zeolites, organoclays, iron-oxide nanocomposites, and alkali-activated materials. By utilizing coral limestone waste as a precursor, CAC offered both high performance and environmental benefits, lowering raw-material costs and minimizing ecological impact. Thus, this approach provided a practical, scalable, and economical adsorbent capable of effectively removing Cr(vi) from aqueous systems.

**Table 6:** Langmuir *q*_max_ (mg g^−1^) for Cr(vi) at 25 °C, pH 2–3 of CAC (current study) *versus* different adsorbents

Adsorbent	pH	*T* (°C)	*Q*_max_ (mg g^−1^)	References
SiO_2_-coated Fe_3_O_4_	2.0	25	13.61	57
ANZ-Fe_3_O_4_	2.0	25	2.85	58
DW/MK-G	2.0	25	50.44	59
AC	2.0	25	5.2	60
*n*Fe_2_O_3_@AC	2.0	25	32.1	60
PGP-1N H_2_SO_4_	3.0	25	28.28	61
Ch-Acs	2.0	25	20.04	62
Acid-treated diatomite	2.0	25	15.01	63
Magnetic biochar	3.0	25	25.27	64
TBC-P	2.0	25	14.97	65
CTAB/H_2_O_2_-clay	2.0	25	67.05	53
CAC	2.0	25	82.93	Current study

### 4.11. Cost analysis and economic probability of CAC manufacture

The economic feasibility of adsorbent materials plays a critical role in their practical application in wastewater treatment systems. The overall production cost encompasses both energy consumption and raw material expenditures. As presented in Table 7, the estimated manufacturing cost of CAC is approximately $0.22 per gram. Moreover, a cost-benefit evaluation demonstrates that CAC provides enhanced economic efficiency relative to traditional treatment technologies and widely used adsorbents, underscoring its potential for cost-effective industrial implementation.

Estimated production cost of CAC derived from coral limestone waste

**Table:** 

Material/chemicals	Purchased quantity	Total purchase cost (USD)	Purchasing cost (USD per g or mL)	Used quantity	Cost of used quantity (USD)
Coral waste	10 kg	—	—	100 g	—
HNO_3_	1 L	10.38 $	0.01 mL	200 mL	2 $
Al(NO_3_)_3_·9H_2_O	500 g	20.31 $	0.04 g	378.6	15.1 $

**Table:** 

Equipment	Time (h)	Max. power (kW)	Unit cost of power	Cost
Calcination	1	1	0.24	0.24 $
Drying	72	1	0.24	17.28 $
Stirrer	2	1	0.24	0.48 $
Total yield cost = 35.1 USD for 158 g			Total yield cost = 0.22 USD per g	

## 5. Conclusions

Calcium aluminate ceramic (CAC) with well-crystallize phases was positively prepared using *Hydnophora* coral biowaste after heat-treatment at 1100 °C. The developed CAC was characterized *via* different analytical methods and used as a bio-based sorbent in the uptake of Cr(vi) ions from solutions. The experimental conditions that controlling the adsorption process were optimized (*i.e.*, pH 2, CAC dose of 200 mg, solution temperature of 55 °C, and shaking time of 2 h). The experimental results were well-fitted by the pseudo-first-order and the Liu models, with maximum uptake capacities between 148.65 and 304.62 mg g^−1^ inside a temperature range of 25–55°. The theoretical treatment organized by the monolayer statistical physics model, which closely matched the experimental data, revealed a multi-docking adsorption mechanism. The density of CAC adsorbent contributed to the endothermic nature of the Cr(vi) removal process and significantly influenced its adsorption capacity. Additionally, the adsorption energies indicated that the Cr(vi) adsorption was primarily driven by physical interactions. Thermodynamics parameters indicated that the Cr(vi)–CAC interface was a spontaneous endothermic physisorption type. The CAC was very stable and suitable for wastewater remediation, according to the regeneration results. In general, utilizing limestone coral biowaste as a low-cost and available feedstock presents a sustainable approach for manufacturing ceramic-based biosorbents for the remediation of metals-contaminated aqueous systems.

## Conflicts of interest

All authors declare that they have no conflicts of interest to disclose.

## Data Availability

The authors declare that the data supporting the findings of this study are available within the article.
